# Defining the Roles of Cardiokines in Human Aging and Age-Associated Diseases

**DOI:** 10.3389/fragi.2022.884321

**Published:** 2022-04-28

**Authors:** Himangi Srivastava, Marina Pozzoli, Edward Lau

**Affiliations:** ^1^ Department of Medicine/Cardiology, School of Medicine, University of Colorado, Aurora, CO, United States; ^2^ Consortium for Fibrosis Research and Translation, School of Medicine, University of Colorado, Aurora, CO, United States

**Keywords:** heart, cardiokine, aging, secretome, proteomics, transcriptomics, hiPSC, endocrine

## Abstract

In recent years an expanding collection of heart-secreted signaling proteins have been discovered that play cellular communication roles in diverse pathophysiological processes. This minireview briefly discusses current evidence for the roles of cardiokines in systemic regulation of aging and age-associated diseases. An analysis of human transcriptome and secretome data suggests the possibility that many other cardiokines remain to be discovered that may function in long-range physiological regulations. We discuss the ongoing challenges and emerging technologies for elucidating the identity and function of cardiokines in endocrine regulations.

## Introduction

The heart is one of the most important organs in terms of age-associated diseases and public health burden in an aging world. An emerging paradigm has focused on how circulatory factors can both contribute to and protect the heart from cardiac pathophysiology in aging and age-related diseases, including circulating growth differentiation factor 8/myostatin (GDF8/MSTN) (Morissette et al., 2009), fibroblast growth factor 21 (FGF21) (Planavila et al., 2015a), midkine (MDK) (Netsu et al., 2014), follistatin-like 3 (FSTL3) (Roh et al., 2019), and others. At the same time, the heart is also an active endocrine organ. Work in the past few decades has established the atrial natriuretic peptide (ANP) and B-type natriuretic peptide (BNP) as critical endocrine signals secreted from the heart. The best understood function of these natriuretic peptides is the regulation of blood pressure and volume via natriuresis and diuresis. However, natriuretic peptide receptors are found in other tissues, where natriuretic peptide signaling can regulate metabolism. In humans, circulating BNP levels increase in age, and are also seen at a higher level in many age-associated conditions including hypertension, suggesting BNP may participate in the regulation of aging-associated processes. More recent studies into the transcript, protein, and secretome expression of heart tissues have found additional secreted proteins to be expressed or secreted from cardiac cells. These molecules have been referred to in the literature as cardiokines and many have been assigned different intercellular communication functions. We consider the current evidence of cardiokine involvement in aging, and discuss ongoing challenges and opportunities in finding cardiokines that may be important in the mechanism of aging processes.

## Definition of Cardiokines

The endocrine function of the heart began to be known with the discovery of the natriuretic peptides in the 1980s (Goetze et al., 2020). References to cardiokines (also referred to as cardiomyokines) first appeared in the literature around 2010, when a number of additional cardiac secreted proteins including mesencephalic astrocyte-derived neurotrophic factor (MANF) were found following seminal work from Glembotski and others (Doroudgar and Glembotski, 2011; Glembotski, 2011; Blackwood et al., 2020). The term has since been applied to a growing set of proteins including proteins found to be secreted from cardiac cells and tissues *in vitro* and *in vivo*. A large complement of these heart secreted proteins has been identified including prominently autocrine and paracrine growth factors, pro-angiogenesis factors, and extracellular matrix remodelers, which have been reviewed comprehensively elsewhere. For the purpose of aging research, here we propose a more limited definition of a *bona fide* cardiokine. In our view, a *bona fide* cardiokine represents a peptide or protein that: 1) is detectable in systemic circulation and has the potential to signal to distal tissues in an endocrine rather than autocrine or paracrine fashion; 2) shows some degree of tissue-biased expression, where an appreciable fraction of a circulating pool is due to secretion from the heart, either in the baseline or in a given diseased condition; and 3) exists as a free extracellular protein or peptide rather than as a cargo of extracellular vesicles, and acts directly on a ligand receptor. This definition of cardiokines is therefore distinguished from the cardiac secretome at large, which includes many local interstitial and matricellular proteins that function in various paracrine and autocrine contexts including a large number of matrix metalloproteinases (MMPs) and tissue inhibitors of metalloproteinases (TIMPs) that function in cardiac extracellular matrix remodeling during aging (Horn and Trafford, 2016). We believe this definition is useful for defining age-associated systemic physiology and is in line with endocrine molecules in pathophysiological processes from other tissues, such as the adipokines and myokines.

### Age-Associated Cardiokine Candidates

We briefly review several cardiokines and cardiokine candidates with the most established current evidence of involvement in age-associated processes in systemic regulation here. Readers are directed to more comprehensive reviews about their pathophysiological functions.

### Atrial Natriuretic Peptide

ANP and BNP are secreted predominantly from the heart and both bind to natriuretic peptide receptors. Beyond the well characterized canonical roles of natriuretic peptides in maintenance of electrolytes, cardio-renal homeostasis, and vasodilation; natriuretic peptide receptors have been found to be present in many tissues, where natriuretic peptide signaling can stimulate fatty acid synthesis in the adipose, regulate fat tissue browning, cell proliferation, and inflammation (Goetze et al., 2020). ANP is primarily secreted from cardiomyocytes in the atrium. ANP level increases with healthy aging (Davis et al., 1996) and also independently with cardiac dysfunction. Increasing levels of ANP in aging therefore is in a prime position to affect systemic physiology in aging.

### B-type Natriuretic Peptide

BNP shares common receptors with ANP but with different affinity, and is primarily secreted from cardiomyocytes in the left ventricle (Yasue et al., 1994). Circulating BNP level increases with healthy aging (Yoshida et al., 2019) as well as heart diseases (Goetze et al., 2020).

### Growth Differentiation Factor 8/Myostatin

GDF8/MSTN is a transforming growth factor beta (TGF-β) family protein that is translated as a 41 kDa pre-prohormone. The signal peptide and prodomains are then cleaved to form a dimer of the 12.5 kDa mature circulating peptide. Both GDF8/MSTN and the closely related growth differentiation factor 11 (GDF11) have been considered putative anti-aging proteins whose decline in age is positively correlated with cardiovascular events and death (Olson et al., 2015). GDF8/MSTN has broad physiological roles in different tissues, including most significantly as an inhibitor of skeletal muscle growth (McPherron et al., 1997), and as a regulator of fibrosis of the heart (Morissette et al., 2009) and mineral density in the bone (Suh et al., 2020). Although GDF8/MSTN is primarily found in skeletal muscle, both fetal and adult hearts express the gene at a low level, and its circulation level is induced following myocardial infarcts (Castillero et al., 2015; Meloux et al., 2019) and heart failure (George et al., 2010). Critically, selective deletion of *Gdf8/Mstn* in the heart is sufficient to prevent muscle loss in mouse models of heart failure (Heineke et al., 2010), thus suggesting the circulating pool of GDF8/MSTN contributed by the heart functions to regulate the mass of skeletal muscles in an endocrine fashion.

### Growth Differentiation Factor 15

GDF15 is a circulating protein involved in the regulation of the body growth and tissue homeostasis, and binds to the GDNF family receptor α–like (GFRAL) receptor (Mullican et al., 2017). GDF15 level increases significantly and prominently with age (Lehallier et al., 2019; Liu et al., 2021) and independently in a variety of disease conditions such as coronary heart diseases (Wollert et al., 2017), myocardial ischemia (Kempf et al., 2006), and heart failure in both humans and mice (Xu et al., 2006). In normal conditions, low levels of expression have been reported in both the skeletal muscle and the hearts of humans and mouse. GDF15 has wide-ranging systemic effects as an endocrine factor; inadequate levels of GDF15 can negatively affect the renal function, whereas overexpression protects from renal and cardiac injuries (Abulizi et al., 2017). Critically, in a mouse model of pediatric dilated cardiomyopathy, increased circulatory GDF15 levels led to decreased body mass and impaired liver growth hormone (GF) signaling, which can be rescued by cardiomyocyte-specific knockdown of *Gdf15*, suggesting GDF15 functions as a heart-to-liver endocrine signal that inhibits body growth in the mouse (Wang et al., 2017).

### Fibroblast Growth Factor 21

FGF21 is translated as a 23 kDa protein in the heparin-binding growth factor family. FGF21 acts as a potent metabolic regulator and stimulates glucose uptake from plasma (Kharitonenkov et al., 2005; Kharitonenkov et al., 2007) and is involved in cardiovascular diseases including heart failure (Chou et al., 2016) and coronary artery diseases (Shen et al., 2013). FGF21 is normally expressed in the liver but is induced in multiple tissues under stress (Keipert and Ost, 2021), and has a relatively short circulating half-life (Kharitonenkov et al., 2007). FGF21 may protect the heart from oxidative stress in an autocrine manner (Planavila et al., 2015b). A prominent increase in measurable circulating levels of FGF21 is seen in human aging (Hanks et al., 2015; Villarroya et al., 2018) as well as in mouse models of cardiac stress (Brahma et al., 2014). Importantly, cardiac-specific overexpression of *Fgf21* increased circulating FGF21 levels in the mouse, and at the same time led to a moderate decrease in body mass, reduction of lean mass, glucose level, and increase in body fat mass (Brahma et al., 2014). This provides evidence for the possibility of cardiac FGF21 acting in an endocrine fashion.

### Other Candidates

Several other cardiokine candidates may function in an endocrine fashion, but it is unclear whether they are associated with aging. For example a cardiac secreted phospholipase A2 has been detected via its activity and that may signal to the liver (Hernandez ‐ Anzaldo et al., 2015). A number of phospholipases A2 family genes have important roles in senescence, but the gene identity of the cardiac secreted isoform(s) is unknown. Cardiomyocyte-specific deletion of follistatin-like 1 (FSTL1) leads to exacerbated renal injuries in experimental models suggesting a potential endocrine effect (Hayakawa et al., 2015), but whether there is any age-associated change is unclear.

Other cardiac secreted proteins are found in significant levels in circulation and may have age-associated expression trends, but so far only their autocrine and paracrine effects have been investigated. MDK is widely expressed and is normally expressed at minimal levels in the heart, but its expression in the heart may be induced by cardiac events (Kitahara et al., 2010). Whether the cardiac-released pool contributes to distal tissue function is unknown. Likewise, FSTL3 acts as an antagonist of TGF-family ligands including probably GDF8/MSTN and GDF11, and its circulation levels in the human plasma, mouse plasma, and transcript expression in the mouse heart are all found to increase with age. However, the effect of cardiac FSTL3 has mostly been studied in its autocrine and paracrine roles.

### Evidence From Large-Scale Human Multi-Omics Data

Upward of 3,000 protein-coding genes, or 15% of the human protein-coding genome, may code for proteins that function at least partially in the extracellular space, and many of them are expressed in the heart at least at the transcript level (Uhlén et al., 2019). Therefore the current number of characterized cardiokines is likely a considerable underestimate of the total number of proteins that are released into circulation from the heart. The increasing power and prevalence of multi-omics technologies offers new approaches to discover currently unknown cardiokines empirically. To estimate the potential number of cardiokine candidates, we queried large-scale human transcriptome data from Genotype-Tissue Expression Project (GTEx) v8. We then sequentially processed, normalized, and batch-corrected the data as previously described (Han et al., 2021). Next, we integrated this data with the circulating protein annotations from the Human Proteome Atlas (HPA) secretome data set (Uhlén et al., 2019), which lists a total of 784 proteins that are annotated as part of the human secretome and moreover was designated to be “secreted to blood” as their location.

**FIGURE 1:**
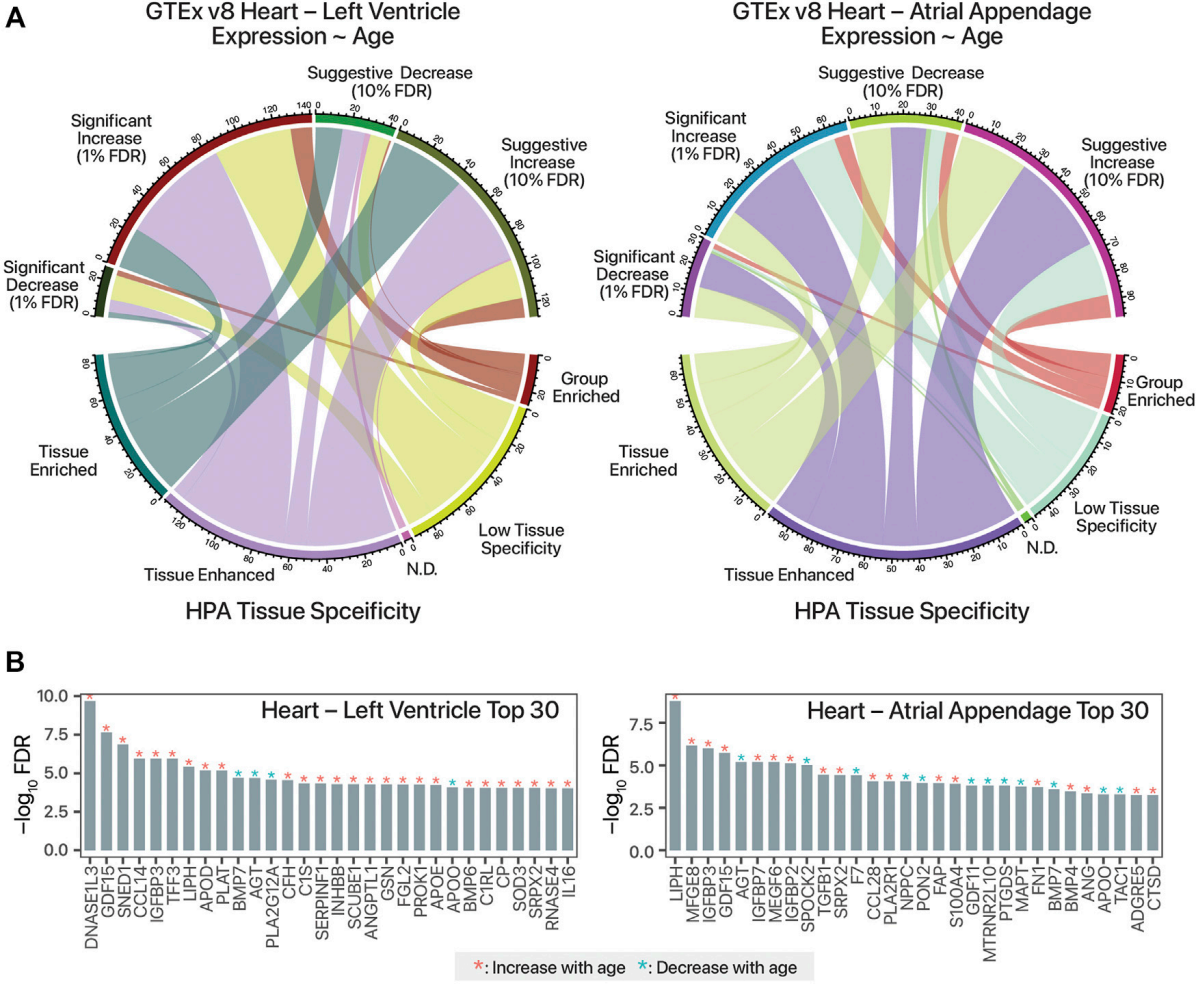
Mining human transcriptomics data for candidate age-associated cardiokines. **(A)**. Chord diagram linking candidate cardiokine-coding transcripts with significant or suggestive age-associated expression trends in humans (Pearson’s correlation test against donor age group *p* value following FDR adjustment of 0.01 and 0.1, respectively) vs. RNA tissue specificity in the Human Protein Atlas (HPA) secretome data set. N.D.: Not detected. **(B)**. Top 30 human secretome atlas protein-coding transcripts that have significant age-associated expression trend in the human heart left ventricle (left; *n* = 432) and atrial appendage (right; *n* = 429) tissues in GTEx v8 are shown. y-axis: log10 of FDR-adjusted *p* value of Pearson’s correlation against age. Red asterisks denote increasing expression trends in older age (r > 0), blue asterisks denote decreasing trends with age (r < 0).

In total, we identified 98 secretome-coding transcripts with age-associated trends in the atrial appendage (68 increasing with age and 30 decreasing); as well as 168 such transcripts in the left ventricle (141 increasing with age and 27 decreasing) (Supplementary Table S1). Relating the GTEx analysis to the HPA secretome data set, it can be seen that the age-differential expressed, candidate cardiokine-encoding transcripts include both those that are tissue-enriched and tissue-enhanced in their expression (Figure 1A). The top age-associated secretome transcripts include those encoding known age-associated circulating factors including GDF15 as well as other known cardiac released factors in both GTEx cardiac tissues (Figure 1B).

**FIGURE 2:**
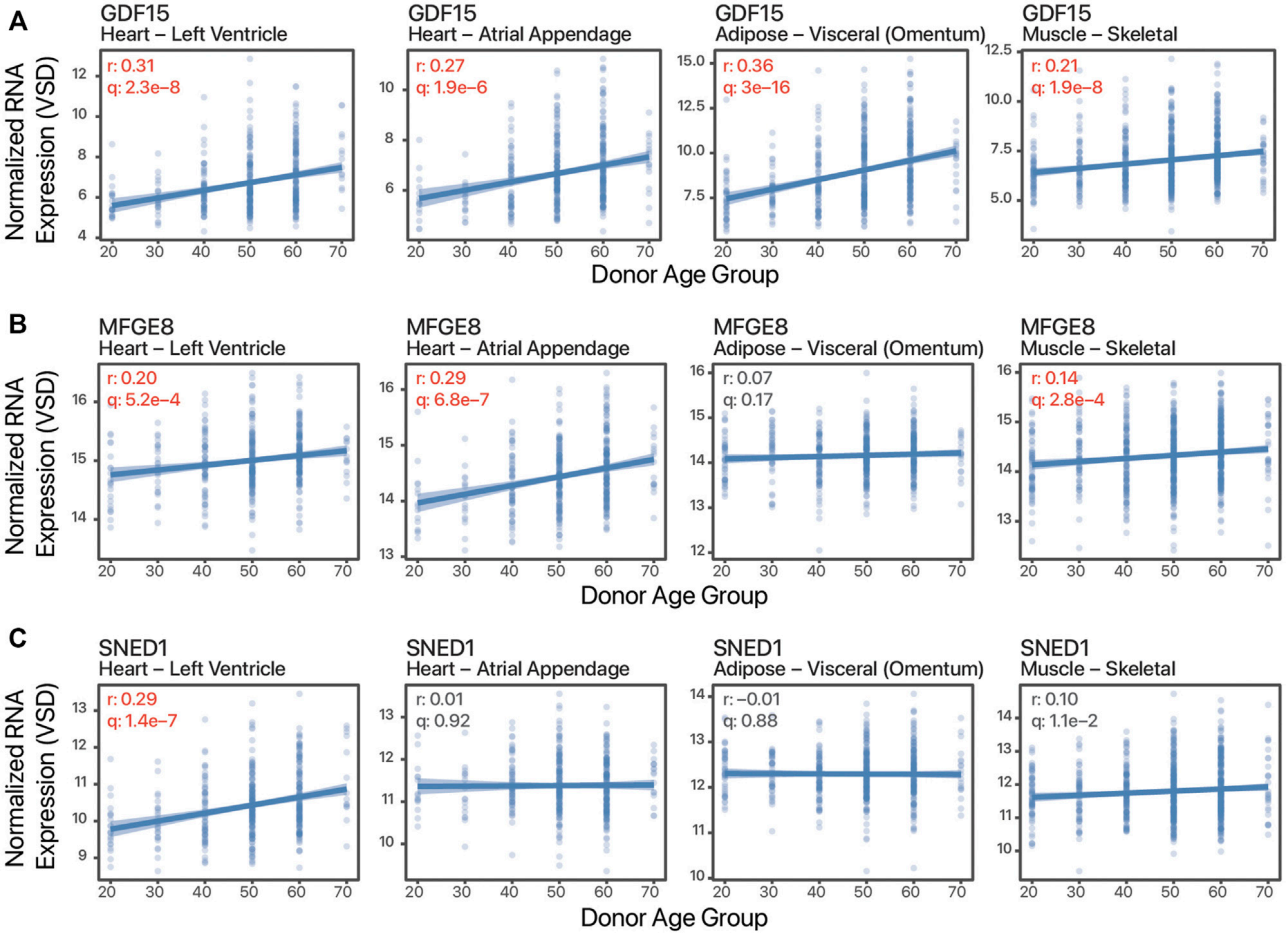
Tissue-biased age-associated expression changes. Example scatter plots of normalized batch-corrected transcript expression against age of three transcripts in four tissues. **(A)**. Growth differentiation factor 15 (GDF15) shows age-associated expression in all four tissues. **(B)**. Milk fat globule-EGF factor 8 protein (MFGE8) shows age-associated increase in the striated muscles but not adipose. **(C)**. Sushi, nidogen, and EGF-like domains 1 (SNED1) shows age-associated increase only in the left ventricle. r: Pearson’s correlation coefficients expression vs. age. q: FDR adjusted *p* value of correlation test. Values in red are significant (q ≤ 0.01).

Besides tissue expression enrichment or specificity, cardiokine-coding transcripts can show tissue bias in age-associated expression changes. These tissue-biased expression changes may reflect pathophysiological cues and signals that are masked in plasma protein level measurements and suggest the possibility of increasing cardiac contributions to the circulating pool in some scenarios. To illustrate, GDF15 has broadly increased expression with age in both cardiac tissues as well as skeletal muscle and adipose samples (Figure 2A), whereas on the other hand, milk fat globule-EGF factor 8 protein (MFGE8) and Sushi, nidogen, and EGF-like domains 1 (SNED1) show age-associated increases only in some tissues but not others (Figure 2B,C). Notwithstanding the obvious caveat of non-correlation between transcript and protein level and between protein level and protein secretion and ligand processing, based on this analysis we surmise that there are likely a number of cardiokine candidates to be discovered whose cardiac-contributed pool in circulation would alter significantly in aging individuals. Further candidate cardiokines may exist that are excluded from the scope of this analysis, for instance, GDF8/MSTN and FSTL3 are both not included within the “secreted to blood” subset of the HPA secretome despite prior literature evidence of their circulatory presence in targeted studies.

### Challenges in Cardiokine Identification and Functional Studies

Given the prevalence of secretome coding transcripts with age-expression trends in cardiac tissues, one might wonder whether more cardiokines stand to be discovered that function in aging. We believe several technical challenges currently exist with regard to the identification of bona fide cardiokines and their role in aging and age-associated diseases.

The discovery of unknown circulatory proteins in the plasma remains difficult. Blood can contain potentially every protein in the human proteome and has a steep dynamic range of concentration which makes it challenging to identify by mass spectrometry. Affinity-based assays have high sensitivity, but their target specificity has been called into question (Joshi and Mayr, 2018). Circulating factors with close sequence homology such as GDF8/MSTN and GDF11 are prone to cross-reactivity (Schafer et al., 2016; Suh and Lee, 2020). Other factors like MDK have multiple splice isoforms, which present additional challenges and may also contribute to undiscovered tissue specificity.

The tissues-of-origin of a protein are difficult to deconvolve from systemic circulation. Plasma is a mixed pool of proteins from different tissues. With possibly the only exceptions of ANP and BNP, cardiac secreted proteins are also released by multiple other tissues, where they likely have different, pleiotropic functions *in situ*. Which tissue dominates as the source of the total circulating pool in the plasma may be dependent on pathophysiological conditions, and a challenge pertains whether the sampled proteins form a single pool or whether the cardiac-released form has distinct function, which may be due to spatiotemporal, concentration-dependent, isoform and proteoform differences. These complications may be partially resolvable by comparing cardiac coronary blood to systemic circulation.

Similarly, it remains a challenge to unravel the target cell and tissue types of signals and associate function. The majority of studies on cardiac secreted proteins focus on characterizing their autocrine/paracrine effects on cardiac physiology and disease responses, hence an age-associated effect in distal tissues may be missed, and this generally contributes to the difficulty of assigning systemic/endocrine physiological functions. Mouse models of cardiac-specific deletion of GDF11 and transgenic expression of GDF15 and MDK exist, but to our knowledge existing reports have only compared their cardiac phenotypes. Large-scale physiological phenotyping comparisons, such as performed on global vs. cardiac-specific GDF8/MSTN knockout mouse model (Morissette et al., 2009; Heineke et al., 2010), are needed. In parallel, multiple circulating signals often share overlapping receptors, for example, ANP and BNP both bind natriuretic peptide receptors, and GDF8/MSTN and GDF11 share activin receptors, hence more isolated systems may be needed to tease apart individual signal contributions.

Lastly, cardiokines are often triggered by myocardial infarct, heart failure, fibrosis, and other age-associated disease conditions (Kitahara et al., 2010). The observation that cardiac secreted proteins increase under stress and disease may be indicative of protective signals in stress or inflammation. Other non-constitutive, cue-specific cardiokines may exist that require targeted characterization in specific conditions to be discovered. More basic studies are thus needed to find out what triggers their release and age-associated change under non-baseline conditions, analogous to exercised induced myokines that have received intense focus for their roles to delay aging processes and promote healthful aging.

### Opportunities and Emerging Approaches

Computational approaches using correlation networks from large-scale sequencing data have been employed to find tissue-specific proteins as well as proteins potentially involved in cross-cell communication. Co-expression analysis is frequently used to identify functionally related genes, based on the assumption that gene pathways may be co-regulated by an unobserved genetic architecture (Szalai and Saez ‐ Rodriguez, 2020). This approach can be extended to multiple transcriptomes to identify unexpected correlation as candidate endocrine signals. An early re-analysis of GTEx data using this approach identified a potential heart to blood signaling protein DPP4, which is known to function in the proteolysis of stromal derived factor 1 (SDF1) (The GTEx Consortium Long et al., 2016). A cross-tissue correlation approach has also been applied to the Hybrid Mouse Diversity Panel (HMDP) data set to find potential endocrine signals between multiple tissues including the liver, adipose, and skeletal muscle (Seldin et al., 2018). Other strategies that make use of cross-transcriptome data may also be instructive, for example, a comparison of tissue transcript levels has been used to predict genes encoding endothelial proteins whose expression across tissue is positively correlated with tissue vascularity, including many secreted proteins (Butler et al., 2016).

Human induced pluripotent stem cells (iPSCs) provide an experimental tool to identify the secretion and downstream signaling function of cardiokines in cells and tissues differentiated from human iPSC lines *in vitro*. The secretome of the resulting cells can then be collected and analyzed using multiple types of omics techniques *in vitro* to discover and confirm secreted proteins, (Hu et al., 2018), or conversely extracellular proteins can be introduced to differentiated cells to assess their downstream effects (Jennbacken et al., 2019). This bottom-up approach circumvents the difficulty of finding the tissue-of-origin from plasma proteins, and can further be used to dissect individual cell type contributions within an organ. Using iPSC differentiation to different lineages, we found different contents in the extracellular vesicles secreted from cardiac myocytes vs. fibroblasts and endothelial cells (Chandy et al., 2020). A similar workflow may be useful for assigning soluble protein cardiokines to different cell types and help elucidate function.

More importantly, multiple cell types from different organs can be simultaneously differentiated that originate from identical iPSC lines and thus carry identical genetic backgrounds, which removes the confounding factors of genetic variance. Engineered “body-on-a-chip” systems containing both iPSC-derived cardiomyocytes and another cell type such as hepatocytes connected by microfluidic devices, for example, have garnered considerable enthusiasm for their potential uses in drug screening and disease modeling (Sharma et al., 2020). Many compounds are metabolized in the liver from pro-drug to active compounds whereas a common side effect of drugs is arrhythmogenicity and cardiotoxicity. Such platforms are being actively explored for the evaluation of adverse effects, and one foresees they can be readily adapted for the purpose of cardiokine studies from cardiac cells to co-cultured cell types as well.

## Concluding Remarks

There is increasing interest in the endocrine functions of the heart and its intersection with human aging and age-associated diseases. Many cardiac secreted proteins are now identified, although thus far few have been established to exist in systemic circulation beyond the cardiac interstitial space, and fewer still have established endocrine signaling functions to distal tissues. Nevertheless, an analysis of human transcriptomics and proteomics data suggests more cardiokines with age-associated expression are likely to be discovered. We believe there is both an opportunity and need to focus on finding long-distance circulatory signals originating from the heart. Future studies may achieve this goal using next-generation stem cell models, multi-omics, and bioinformatics techniques.

## References

[B1] Abulizi P. Loganathan N. Zhao D. Mele T. Zhang Y. Zwiep T. (2017). Growth Differentiation Factor-15 Deficiency Augments Inflammatory Response and Exacerbates Septic Heart and Renal Injury Induced by Lipopolysaccharide. Sci. Rep. 7 (1), 1037. 10.1038/s41598-017-00902-5 28432312 PMC5430818

[B2] Blackwood E. A. Thuerauf D. J. Stastna M. Stephens H. Sand Z. Pentoney A. (2020). Proteomic Analysis of the Cardiac Myocyte Secretome Reveals Extracellular Protective Functions for the ER Stress Response. J. Mol. Cell Cardiol. 143, 132–144. 10.1016/j.yjmcc.2020.04.012 32339566 PMC8597053

[B3] Brahma M. K. Adam R. C. Pollak N. M. Jaeger D. Zierler K. A. Pöcher N. (2014). Fibroblast Growth Factor 21 Is Induced upon Cardiac Stress and Alters Cardiac Lipid Homeostasis. J. Lipid Res. 55 (11), 2229–2241. 10.1194/jlr.M044784 25176985 PMC4617126

[B4] Butler L. M. Hallström B. M. Fagerberg L. Pontén F. Uhlén M. Renné T. (2016). Analysis of Body-wide Unfractionated Tissue Data to Identify a Core Human Endothelial Transcriptome. Cell Syst 3 (3), 287–301. e3. 10.1016/j.cels.2016.08.001 27641958

[B5] Castillero E. Akashi H. Wang C. Najjar M. Ji R. Kennel P. J. (2015). Cardiac Myostatin Upregulation Occurs Immediately after Myocardial Ischemia and Is Involved in Skeletal Muscle Activation of Atrophy. Biochem. Biophysical Res. Commun. 457 (1), 106–111. 10.1016/j.bbrc.2014.12.057 PMC4967536 25528587

[B6] Chandy M. Rhee J.-W. Ozen M. O. Williams D. R. Pepic L. Liu C. (2020). Atlas of Exosomal MicroRNAs Secreted from Human IPSC-Derived Cardiac Cell Types. Circulation 142 (18), 1794–1796. 10.1161/CIRCULATIONAHA.120.048364 33136510 PMC8135104

[B7] Chou R.-H. Huang P.-H. Hsu C.-Y. Chang C.-C. Leu H.-B. Huang C.-C. (2016). Circulating Fibroblast Growth Factor 21 Is Associated with Diastolic Dysfunction in Heart Failure Patients with Preserved Ejection Fraction. Sci. Rep. 6, 33953. 10.1038/srep33953 27650781 PMC5030655

[B8] Davis K. M. Fish L. C. Minaker K. L. Elahi D. (1996). Atrial Natriuretic Peptide Levels in the Elderly: Differentiating Normal Aging Changes from Disease. Journals Gerontol. Ser. A: Biol. Sci. Med. Sci. 51A (3), M95–M101. 10.1093/gerona/51A.3.M95 8630708

[B9] Doroudgar S. Glembotski C. C. (2011). The Cardiokine Story Unfolds: Ischemic Stress-Induced Protein Secretion in the Heart. Trends Mol. Med. 17 (4), 207–214. 10.1016/j.molmed.2010.12.003 21277256 PMC3078974

[B10] George I. Bish L. T. Kamalakkannan G. Petrilli C. M. Oz M. C. Naka Y. (2010). Myostatin Activation in Patients with Advanced Heart Failure and after Mechanical Unloading. Eur. J. Heart Fail. 12 (5), 444–453. 10.1093/eurjhf/hfq039 20348550 PMC2857990

[B11] Glembotski C. C. (2011). Functions for the Cardiomyokine, MANF, in Cardioprotection, Hypertrophy and Heart Failure. J. Mol. Cell Cardiol. 51 (4), 512–517. 10.1016/j.yjmcc.2010.10.008 20970425 PMC3660035

[B12] Goetze J. P. Bruneau B. G. Ramos H. R. Ogawa T. de Bold M. K. de Bold A. J. (2020). Cardiac Natriuretic Peptides. Nat. Rev. Cardiol. 17 (11), 698–717. 10.1038/s41569-020-0381-0 32444692

[B13] Han Y. Li L. Z. Kastury N. L. Thomas C. T. Lam M. P. Y. Lau E. (2021). Transcriptome Features of Striated Muscle Aging and Predictability of Protein Level Changes. Mol. Omics 17, 796–808. 10.1039/d1mo00178g 34328155 PMC8511094

[B14] Hanks L. J. Gutiérrez O. M. Bamman M. M. Ashraf A. McCormick K. L. Casazza K. (2015). Circulating Levels of Fibroblast Growth Factor-21 Increase with Age Independently of Body Composition Indices Among Healthy Individuals. J. Clin. Translational Endocrinol. 2 (2), 77–82. 10.1016/j.jcte.2015.02.001 PMC4450097 26042208

[B15] Hayakawa S. Ohashi K. Shibata R. Kataoka Y. Miyabe M. Enomoto T. (2015). Cardiac Myocyte-Derived Follistatin-like 1 Prevents Renal Injury in a Subtotal Nephrectomy Model. Jasn 26 (3), 636–646. 10.1681/ASN.2014020210 25071081 PMC4341480

[B16] Heineke J. Auger-Messier M. Xu J. Sargent M. York A. Welle S. (2010). Genetic Deletion of Myostatin from the Heart Prevents Skeletal Muscle Atrophy in Heart Failure. Circulation 121 (3), 419–425. 10.1161/CIRCULATIONAHA.109.882068 20065166 PMC2823256

[B17] Hernandez‐Anzaldo S. Berry E. Brglez V. Leung D. Yun T. J. Lee J. S. (2015). Identification of a Novel Heart-Liver Axis: Matrix Metalloproteinase‐2 Negatively Regulates Cardiac Secreted Phospholipase A 2 to Modulate Lipid Metabolism and Inflammation in the Liver. Jaha 4 (11). 10.1161/JAHA.115.002553 PMC4845223 26567374

[B18] Horn M. A. Trafford A. W. (2016). Aging and the Cardiac Collagen Matrix: Novel Mediators of Fibrotic Remodelling. J. Mol. Cell Cardiol. 93, 175–185. 10.1016/j.yjmcc.2015.11.005 26578393 PMC4945757

[B19] Hu N.-W. Corbett G. T. Moore S. Klyubin I. O’Malley T. T. Walsh D. M. (2018). Extracellular Forms of Aβ and Tau from IPSC Models of Alzheimer’s Disease Disrupt Synaptic Plasticity. Cell Rep 23 (7), 1932–1938. 10.1016/j.celrep.2018.04.040 29768194 PMC5972225

[B20] Jennbacken K. Wågberg F. Karlsson U. Eriksson J. Magnusson L. Chimienti M. (2019). Phenotypic Screen with the Human Secretome Identifies FGF16 as Inducing Proliferation of IPSC-Derived Cardiac Progenitor Cells. Int. J. Mol. Sci. 20 (23), 6037. 10.3390/ijms20236037 PMC6928864 31801200

[B21] Joshi A. Mayr M. (2018). In Aptamers They Trust. Circulation 138 (22), 2482–2485. 10.1161/CIRCULATIONAHA.118.036823 30524136 PMC6277005

[B22] Keipert S. Ost M. (2021). Stress-Induced FGF21 and GDF15 in Obesity and Obesity Resistance. Trends Endocrinol. Metab. 32 (11), 904–915. 10.1016/j.tem.2021.08.008 34526227

[B23] Kempf T. Eden M. Strelau J. Naguib M. Willenbockel C. Tongers J. (2006). The Transforming Growth Factor-β Superfamily Member Growth-Differentiation Factor-15 Protects the Heart from Ischemia/Reperfusion Injury. Circ. Res. 98 (3), 351–360. 10.1161/01.RES.0000202805.73038.48 16397141

[B24] Kharitonenkov A. Shiyanova T. L. Koester A. Ford A. M. Micanovic R. Galbreath E. J. (2005). FGF-21 as a Novel Metabolic Regulator. J. Clin. Invest. 115 (6), 1627–1635. 10.1172/JCI23606 15902306 PMC1088017

[B25] Kharitonenkov A. Wroblewski V. J. Koester A. Chen Y.-F. Clutinger C. K. Tigno X. T. (2007). The Metabolic State of Diabetic Monkeys Is Regulated by Fibroblast Growth Factor-21. Endocrinology 148 (2), 774–781. 10.1210/en.2006-1168 17068132

[B26] Kitahara T. Shishido T. Suzuki S. Katoh S. Sasaki T. Ishino M. (2010). Serum Midkine as a Predictor of Cardiac Events in Patients with Chronic Heart Failure. J. Card. Fail. 16 (4), 308–313. 10.1016/j.cardfail.2009.12.014 20350697

[B27] Lehallier B. Gate D. Schaum N. Nanasi T. Lee S. E. Yousef H. (2019). Undulating Changes in Human Plasma Proteome Profiles across the Lifespan. Nat. Med. 25 (12), 1843–1850. 10.1038/s41591-019-0673-2 31806903 PMC7062043

[B28] Liu H. Huang Y. Lyu Y. Dai W. Tong Y. Li Y. (2021). GDF15 as a Biomarker of Ageing. Exp. Gerontol. 146, 111228. 10.1016/j.exger.2021.111228 33421539

[B30] McPherron A. C. Lawler A. M. Lee S.-J. (1997). Regulation of Skeletal Muscle Mass in Mice by a New TGF-P Superfamily Member. Nature 387 (6628), 83–90. 10.1038/387083a0 9139826

[B31] Meloux A. Rochette L. Maza M. Bichat F. Tribouillard L. Cottin Y. (2019). Growth Differentiation Factor-8 (GDF8)/Myostatin Is a Predictor of Troponin I Peak and a Marker of Clinical Severity after Acute Myocardial Infarction. Jcm 9 (1), 116. 10.3390/jcm9010116 PMC7019567 31906236

[B32] Morissette M. R. Stricker J. C. Rosenberg M. A. Buranasombati C. Levitan E. B. Mittleman M. A. (2009). Effects of Myostatin Deletion in Aging Mice. Aging Cell 8 (5), 573–583. 10.1111/j.1474-9726.2009.00508.x 19663901 PMC2764272

[B33] Mullican S. E. Lin-Schmidt X. Chin C.-N. Chavez J. A. Furman J. L. Armstrong A. A. (2017). GFRAL Is the Receptor for GDF15 and the Ligand Promotes Weight Loss in Mice and Nonhuman Primates. Nat. Med. 23 (10), 1150–1157. 10.1038/nm.4392 28846097

[B34] Netsu S. Shishido T. Kitahara T. Honda Y. Funayama A. Narumi T. (2014). Midkine Exacerbates Pressure Overload-Induced Cardiac Remodeling. Biochem. Biophysical Res. Commun. 443 (1), 205–210. 10.1016/j.bbrc.2013.11.083 24291499

[B35] Olson K. A. Beatty A. L. Heidecker B. Regan M. C. Brody E. N. Foreman T. (2015). Association of Growth Differentiation Factor 11/8, Putative Anti-ageing Factor, with Cardiovascular Outcomes and Overall Mortality in Humans: Analysis of the Heart and Soul and HUNT3 Cohorts. Eur. Heart J. 36 (48), 3426–3434. 10.1093/eurheartj/ehv385 26294790 PMC4685178

[B36] Planavila A. Redondo-Angulo I. Ribas F. Garrabou G. Casademont J. Giralt M. (2015). Fibroblast Growth Factor 21 Protects the Heart from Oxidative Stress. Cardiovasc. Res. 106 (1), 19–31. 10.1093/cvr/cvu263 25538153

[B37] Planavila A. Redondo-Angulo I. Villarroya F. (2015). FGF21 and Cardiac Physiopathology. Front. Endocrinol. 6. 10.3389/fendo.2015.00133 PMC4553397 26379627

[B38] Roh J. D. Hobson R. Chaudhari V. Quintero P. Yeri A. Benson M. (2019). Activin Type II Receptor Signaling in Cardiac Aging and Heart Failure. Sci. Transl. Med. 11 (482), eaau8680. 10.1126/scitranslmed.aau8680 30842316 PMC7124007

[B39] Schafer M. J. Atkinson E. J. Vanderboom P. M. Kotajarvi B. White T. A. Moore M. M. (2016). Quantification of GDF11 and Myostatin in Human Aging and Cardiovascular Disease. Cel Metab. 23 (6), 1207–1215. 10.1016/j.cmet.2016.05.023 PMC4913514 27304512

[B40] Seldin M. M. Koplev S. Rajbhandari P. Vergnes L. Rosenberg G. M. Meng Y. (2018). A Strategy for Discovery of Endocrine Interactions with Application to Whole-Body Metabolism. Cell Metab 27 (5), 1138–1155. e6. 10.1016/j.cmet.2018.03.015 29719227 PMC5935137

[B41] Sharma A. Sances S. Workman M. J. Svendsen C. N. (2020). Multi-Lineage Human IPSC-Derived Platforms for Disease Modeling and Drug Discovery. Cell Stem Cell 26 (3), 309–329. 10.1016/j.stem.2020.02.011 32142662 PMC7159985

[B42] Shen Y. Ma X. Zhou J. Pan X. Hao Y. Zhou M. (2013). Additive Relationship between Serum Fibroblast Growth Factor 21 Level and Coronary Artery Disease. Cardiovasc. Diabetol. 12, 124. 10.1186/1475-2840-12-124 23981342 PMC3766150

[B43] Suh J. Kim N.-K. Lee S.-H. Eom J.-H. Lee Y. Park J.-C. (2020). GDF11 Promotes Osteogenesis as Opposed to MSTN, and Follistatin, a MSTN/GDF11 Inhibitor, Increases Muscle Mass but Weakens Bone. Proc. Natl. Acad. Sci. U.S.A. 117 (9), 4910–4920. 10.1073/pnas.1916034117 32071240 PMC7060712

[B44] Suh J. Lee Y.-S. (2020). Similar Sequences but Dissimilar Biological Functions of GDF11 and Myostatin. Exp. Mol. Med. 52 (10), 1673–1693. 10.1038/s12276-020-00516-4 33077875 PMC8080601

[B45] Szalai B. Saez‐Rodriguez J. (2020). Why Do Pathway Methods Work Better Than They Should? FEBS Lett. 594 (24), 4189–4200. 10.1002/1873-3468.14011 33270910

[B29] The GTEx Consortium Long Q. Argmann C. Argmann C. Houten S. M. Huang T. Peng S. (2016). Inter-Tissue Coexpression Network Analysis Reveals DPP4 as an Important Gene in Heart to Blood Communication. Genome Med. 8 (1), 15. 10.1186/s13073-016-0268-1 26856537 PMC4746932

[B46] Uhlén M. Karlsson M. J. Hober A. Svensson A.-S. Scheffel J. Kotol D. (2019). The Human Secretome. Sci. Signal. 12 (609), eaaz0274. 10.1126/scisignal.aaz0274 31772123

[B47] Villarroya J. Gallego-Escuredo J. M. Delgado-Anglés A. Cairó M. Moure R. Gracia Mateo M. (2018). Aging Is Associated with Increased FGF21 Levels but Unaltered FGF21 Responsiveness in Adipose Tissue. Aging Cell 17 (5), e12822. 10.1111/acel.12822 30043445 PMC6156525

[B48] Wang T. Liu J. McDonald C. Lupino K. Zhai X. Wilkins B. J. (2017). GDF 15 Is a Heart‐derived Hormone that Regulates Body Growth. EMBO Mol. Med. 9 (8), 1150–1164. 10.15252/emmm.201707604 28572090 PMC5538424

[B49] Wollert K. C. Kempf T. Wallentin L. (2017). Growth Differentiation Factor 15 as a Biomarker in Cardiovascular Disease. Clin. Chem. 63 (1), 140–151. 10.1373/clinchem.2016.255174 28062617

[B50] Xu J. Kimball T. R. Lorenz J. N. Brown D. A. Bauskin A. R. Klevitsky R. (2006). GDF15/MIC-1 Functions as a Protective and Antihypertrophic Factor Released from the Myocardium in Association with SMAD Protein Activation. Circ. Res. 98 (3), 342–350. 10.1161/01.RES.0000202804.84885.d0 16397142

[B51] Yasue H. Yoshimura M. Sumida H. Kikuta K. Kugiyama K. Jougasaki M. (1994). Localization and Mechanism of Secretion of B-type Natriuretic Peptide in Comparison with Those of A-type Natriuretic Peptide in Normal Subjects and Patients with Heart Failure. Circulation 90 (1), 195–203. 10.1161/01.cir.90.1.195 8025996

[B52] Yoshida Y. Nakanishi K. Daimon M. Ishiwata J. Sawada N. Hirokawa M. (2019). Alteration of Cardiac Performance and Serum B-type Natriuretic Peptide Level in Healthy Aging. J. Am. Coll. Cardiol. 74 (14), 1789–1800. 10.1016/j.jacc.2019.07.080 31582139

